# The Clinical Application of EEG-Signals Recurrence Analysis as a Measure of Functional Connectivity: Comparative Case Study of Patients with Various Neuropsychiatric Disorders

**DOI:** 10.3390/brainsci10060380

**Published:** 2020-06-16

**Authors:** Kamil Jonak, Arkadiusz Syta, Hanna Karakuła-Juchnowicz, Paweł Krukow

**Affiliations:** 1 Department of Psychiatry, Psychotherapy and Early Intervention, Medical University of Lublin, 20-439 Lublin, Poland; karakula.hanna@gmail.com; 2 Mechanical Engineering Faculty, Lublin University of Technology, Nadbystrzycka 38 D, 20-618 Lublin, Poland; a.syta@pollub.pl; 3 Department of Clinical Neuropsychiatry, Medical University of Lublin, ul. Gluska 1, 20-439 Lublin, Poland; pawelkrukow@umlub.pl

**Keywords:** neuroinformatics, non-linear time series analysis, EEG, schizophrenia, white matter

## Abstract

Background. An electroencephalogram (EEG) is a simple and widely used assessment tool that allows one to analyze the bioelectric activity of the brain. As a result, one can observe brain waves with different frequencies and amplitudes that correspond to the temporary synchronization of different parts of the brain. Synchronization patterns may be changed by almost any type of pathological conditions, such as psychiatric diseases and structural abnormalities of the brain tissue. In various neuropsychiatric disorders, the coordination of cortical activity may be decreased or enhanced as a result of neurobiological compensatory mechanisms. Methods. In this paper, we analyzed the EEG signals in resting-state condition, with reference to three patients with a similar set of psychopathological symptoms typical for the first psychotic episode, but with different functional and structural neural basis of the disease. Additionally, those patients were compared with a demographically matched healthy individual. We used the non-linear method of time series analysis based on the recurrences of states, to verify whether functional connectivity configurations assessed with recurrence method will qualitatively distinguish patients from a healthy subject, but also differentiate patients from each other. Results. Obtained results confirmed that the connectivity architecture mapped with the recurrence analysis substantially differentiated all participants from each other. An applied analysis additionally showed the specificity of cortical desynchronization and over-synchronization matched to the psychiatric or neurological basis of the disease. Despite this encouraging finding, group-oriented studies are needed to corroborate our qualitative results, based only on a series of clinical case studies.

## 1. Introduction

Actual studies on the neurobiological underpinning of schizophrenia are focused on describing specific patterns of structural and functional connectivity, defined as an anatomical and dynamical basis of brain coordination. This approach originates in a well-documented theory, according to which schizophrenia is a disconnection disorder, i.e., the observed psychiatric symptoms, as well as the co-occurring cognitive disorder, result from the disconnection of various brain systems regulating the activity of the mind [1,2,3]. The disconnection hypothesis is strongly associated with a substantial increase in the number of publications confirming the occurrence of abnormalities concerning the white matter (WM), ensuring the coordination of various cortical areas and subcortical gray matter nuclei [4]. These WM abnormalities in the majority of schizophrenic patients involve microstructural lesions, including myelin damage, gliosis, and tissue edema, which are not observable in simple anatomical magnetic resonance imaging (MRI) protocols (i.e., T1), but can be captured only using diffusion tensor imaging (DTI) MRI sequences [5,6]. Correlations between WM disorders and the clinical picture of psychosis were also noticed in the form of relatively rare neurological diseases, in which the progressive damage of large axonal fibers was present, with simultaneously occurring neuropsychiatric symptoms similar to those observed in the development of schizophrenia. The WM disease most often confused with schizophrenia is metachromatic leukodystrophy (ML). ML affecting children and young adults is caused by a deficiency of arylsulphatase A (ARSA) and an accumulation of metachromatic sulphatide deposits within the nervous system and internal organs [7]. The existence of so-called schizophrenia-like neurological disorders suggests a blurring of the boundary between endogenous psychotic diseases and quasi-psychiatric neurological conditions, which, due to their etiology, should be treated in distinct manners, and will also significantly differ in the clinical prognosis. Despite substantial effort to establish the biomarkers of psychiatric disorders allowing differential diagnosis based on an objective indicator, such biomarkers have not yet emerged. It justifies further attempts to find specific features of the brain functioning typical for subjects with endogenous psychosis, patients with neurological disorders with a clinical picture similar to schizophrenia and healthy individuals [8].

Considering the above, the aim of this study is to analyze specific features of functional connectivity, tested on the basis of a resting-state electroencephalogram (EEG) records from four different subjects: a healthy person, patient diagnosed with schizophrenia without any changes in the WM present in MRI scan, patient diagnosed with schizophrenia and co-occurring structural WM lesions, visible in the classic MRI and patient, also with structural WM lesions, in which schizophrenia was initially diagnosed on the basis of the clinical picture, however, further assessment and development of neuropsychiatric symptoms indicated the neurological background of the disease. Functional connectivity emanated from resting-state EEG refers to processes of temporal synchronization between given brain regions, based on several features of the electrophysiological signal [9,10]. This type of connectivity concerns neuronal coordination which does not have to depend on WM fibers, however, WM damage might be reflected at the functional connectivity level by the overcompensation of synchronization at the cortical level, induced by the necessity to maintain connectivity between different cortical areas, despite the interruption of a long axonal fiber [11]. Brain activity can be compared to the state of a nonlinear dynamic system, which at a given moment of time, is clearly determined by the so-called coordinates of the phase space describing its evolution in time. In our work, the role of the space of all possible states is played by the recorded EEG time series using the electro-cap. Each of the registered channels carries information about brain dynamics, and correlation (synchronization) between them may vary depending on the disease case. The linear relationship between EEG channels is often studied using correlation and coherence, which is relatively easy to apply, but they do not take into account nonlinear relationships [12]. Analyzing the non-linear correlations between EEG signals can be helpful to detect differences that were not visible to linear methods, and that is why we propose a method of recurrences [13,14]. This method involves testing similarities between individual states of the system and was previously used to analyze functional connectivity. For example, Acharya [15] analyzed the non-linear dynamics of the brain in different sleep phases and found that its synchronization decreases in phases 0–4 and increases in phase 5. Rangaprakash [16], using recurrence indicators, studied patients with global and local epilepsy, by designing maps of synchronization between EEG channels. Based on this approach, it was demonstrated that patients with global epilepsy showed intensified synchronization in virtually the entire cerebral cortex, while patients with focal epilepsy showed this only in selected areas. In other studies [17,18], the authors also used the method of non-linear recurrence analysis to detect different epilepsy states in patients and proved the clinical usefulness of the recurrence analysis for processing EEG data. In the paper by Stam [19], one can find a comprehensive review with examples on the use of non-linear analysis methods to EEG-data in the case of various diseases and therapies. Recently, Lombardi and others [20] have successfully applied the recurrence analysis method to study changes in brain dynamics in nine patients with schizophrenia and compared them with a nine-person control group during a working memory task. It turned out that by analyzing the periodicity in the considered time series of functional MRI (fMRI), they were able to indicate the difference in the selected recurrence rates.

The main purpose of the work is a non-linear analysis of the correlation between EEG channels, which could act as a measure of synchronization between brain areas and distinguish various cases of illness by the value of certain quantifiers. In other words, one might assume that various features of functional connectivity measured with recurrence analysis (nonlinear correlations) in our four individuals enable the registration of specific parameters of neural synchronization, that will qualitatively distinguish a healthy individual from patients with neuropsychiatric disorders, but also patients with a similar clinical picture of psychiatric disorders, but a different neurobiological background. It is worth mentioning that fMRI testing is quite expensive and more difficult to carry out than the EEG that we used in this study.

## 2. Materials and Methods

### 2.1. Cases

**Figure 1:**
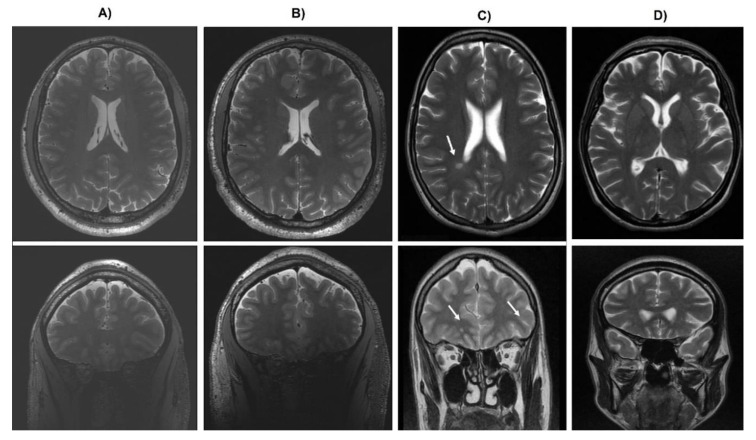
T2 Magnetic resonance images captured from (**A**) health participant (H), (**B**) participant with diagnosed schizophrenia (S), (**C**) patient with white matter hyperintensities (SN), (**D**) patient (N).

As stated earlier, four subjects participated in this study. (a) A healthy woman, aged 24, with a university degree, without any psychiatric and neurological diseases (H), (b) 25 aged woman with a university degree, diagnosed with schizophrenia based on Diagnostic and Statistical Manual of Mental Disorders, 5th Edition (DSM-5) criteria, without comorbid neurological disorders assessed in a clinical evaluation and with an MRI result without any structural abnormalities (S). The patients were treated with a 20 mg daily dose of Olanzapine during EEG recording. Based on psychiatric assessment, the patient (b) responded well to the applied antipsychotic treatment, and as a result of pharmacotherapy, obtained the level of clinical remission characteristic for patients with schizophrenia. The psychopathological picture of patient b was dominated by persecutory delusions and formal thoughts disorder, in a form of atypical semantic associations. This symptoms significantly improved after about 4–6 weeks of antipsychotic treatment. Third subject (c), was a 23 year old woman, a third year student of humanities, diagnosed with schizophrenia based on DSM-5 criteria (SN). During the routine MRI (1.5 T) examination, the following structural abnormalities, including the white matter, have been revealed: more than seven foci of white matter hyperintensities seen on MRI images, including fluid attenuated inversion recovery sequence imaging present in both hemispheres, located in deep white matter and periventricular, size up to 8 mm. Neuroradiologist’s judgment did not indicate a clear etiology of the above changes; at the same time, it was indicated that these abnormalities were not the basis of the psychiatric disorders present in the patient. The patient was also treated with 20 mg of Olanzapine daily and her psychopathological state significantly improved after the standard antipsychotic therapy. As in the previous case (patient b), formal thoughts disorders were present as one of the main psychopathological syndrome, accompanied by a lack of initiative and a tendency to socially isolate. Patient (c) was consulted by a neurologist, the clinical examination revealed no deviations from the norm, additionally, she was tested for Lyme disease (infection caused by the spirochete Borrelia burgdorferi), however, negative test results were obtained. Due to the lack of an effect-related associations between the occurrence of psychiatric symptoms and changes in structural neuroimaging, the appearance of a clinical picture typical for schizophrenia, and the significant improvement obtained as a result of standard psychiatric treatment, this patient was qualified by us as a person diagnosed as schizophrenic with coexisting abnormalities in the white matter. The last participant (d) was a 28-year-old woman (N), a second year student of humanities, with a preliminary diagnosis of schizophrenia, based mainly on symptoms similar to those classified as the “negative” syndromes of schizophrenia (lack of initiative, reduced affect, social withdrawal, and deeply reduced spontaneous behaviors and verbal communications [21]), however, symptoms such as hallucinations or delusions have not been observed. These patients finally appeared to have a neurological disorder caused by an infectious disease of the central nervous system, only manifested with neuropsychiatric symptoms. The following facts suggested the primary neurological aetiology of the disorders: persistence of neuropsychiatric symptoms, despite taking many forms of treatment with various type antipsychotics, the results of cerebrospinal fluid and blood biochemical tests confirming inflammation within the nervous system, progressively increasing symptoms of motoric retardation and disorders of verbal communication in a form of non-fluent speech, organic eating disorders, the occurrence of a paradoxical reaction to antipsychotic drugs in the form of delirium, a strictly subcortical profile of severe cognitive dysfunctions, the result of a neuroimaging assessment with the statement of the most likely post-inflammatory lesions of white matter. The white matter lesions consisted in many hyperintensive changes located in the subcortical white matter of the frontal lobe and parietal area (see Figure 1). The biological factor (a virus or bacteria) that triggered inflammation was not finally identified, because it was active long before the admission to the psychiatric ward. The EEG assessment took place in the last week of hospitalization, when the patient was treated with a low dose of atypical antipsychotics, which was well tolerated by the patient (4 mg/d of Risperidone). All patients were recruited from the I Department of Psychiatry, Psychotherapy and Early Intervention, Medical University of Lublin, Poland, and provided informed consent for a protocol approved by the Bioethical Commission of the Medical University of Lublin. In addition, all the methods used in the study were performed in accordance with all the relevant guidelines and regulations of the Bioethical Commission.

### 2.2. EEG Data Recording

In all cases, eight minutes of resting state EEG data was recorded using a 21 scalp location electro-cap (Electro-Cap International Inc., Eaton, OH, USA) and Ag/AgCl disk electrodes with an average common reference. Electrodes have been deployed according to 10–20 international systems (Fp1, Fp2, F3, F4, C3, C4, P3, P4, O1, O2, A1, A2, F7, F8, T3, T4, T5, T6, Fz, Pz and Cz). During the recordings, impedance value of the electrodes was kept below 5 kΩ, with the sampling rate of 512 Hz and an active notch filter, set at 50 Hz. After having been recorded, the data were exported to ASCII format. For further analysis, the ASCII files were imported to MATLAB (Mathworks Inc. 3 Apple Hill Drive Natick, Massachusetts), band-pass filtered from 0.5 to 45 Hz and split into 2500 long epochs. It is worth noting that we did not split the epochs for frequency components corresponding to particular bands, but we used a one-dimensional time series that corresponded to the EEG record of a single electrode. Thus, each patient with different features of functional connectivity (H, SN, S and N) was characterized by four time series of equal length without artifacts and all results of the analyzes carried out were obtained as an average for four cut out signals. For comparison between individual patients, each of the time series was normalized with a mean of 0 and a standard deviation of 1.

### 2.3. EEG Recurrence Analysis

**Figure 2:**
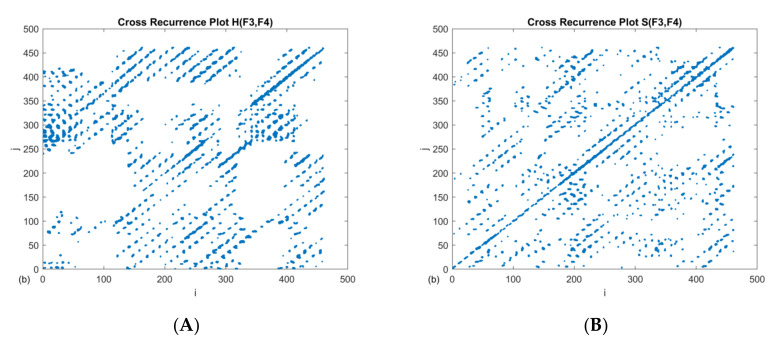
Recurrence plot RP between two electrodes (F3-F4) of filtered and normalized EEG signal for: (**A**) healthy patient, (**B**) patient with schizophrenic. In both cases, same embedding has been used (*τ* = 10, *m* = 5) and same threshold *ε* = 1.1.

The recurrence method was originally proposed by Eckmann [22], who observed that non-linear complex dynamical systems have the property of repeatability (recurrence). This means that the evolution of a given system at a specific moment of a time is very similar to the evolution at the moment that occurred earlier (most often a certain period). Such an event is what Eckmann called recurrence in nonlinear dynamical systems, which can be illustrated by two points close enough to each other on the phase trajectory given by the state space coordinates that were mentioned earlier. The state vector coordinates can be determined using a mathematical model or experimental data. In the second case, it is not always possible to register all coordinates, but you can reconstruct the missing coordinates from a single observation. The proper reconstruction of a phase space is crucial for the whole procedure and will be briefly described after Takens [23]. First of all, let us denote $\vec{x\left(t\right)}=\left(x_{1}\left(t\right),x_{2}\left(t\right),\ldots ,x_{d}\left(t\right)\right)$ as a state of the system in *d* dimensional state space in time. The coordinates of $\vec{x\left(t\right)}$ are coupled in a sense that each of them possess the information about dynamics of the whole system. Therefore, one can reconstruct the topological equivalent phase space $\vec{\tilde{x}\left(t\right)}$ under the assumption that the system is smooth enough (its mathematical model). In that case,

$$\vec{\tilde{x}\left(t\right)}=\left(x_{i}\left(t\right),x_{i}\left(t-\tau \right),x_{i}\left(t-2\tau \right)\ldots ,x_{i}\left(t-2m\tau \right)\right)$$

where *τ* is a time delay and *m* is an embedding dimension. The time delay value should be optimal, so that the coordinates are correlated with each other in both the short and long time scale. Time delay can be found by the mutual information method, that takes into account nonlinear correlations between data points [24] (the first minima is often taken as *τ*, here *τ* = 10). Moreover, the value of the embedding dimension should be properly selected, so that two nearby points do not turn out to be distant from each other in a space with a higher dimension. The embedding dimension can be chosen by using the false nearest neighbours method [25] (the zero of this function can be taken as a *m*, here *m* = 5). The chosen values of the embedding parameters were determined as the median of the calculated values for each individual electrode. After proper reconstruction of the systems phase space, one can apply the method of recurrences and calculate the recurrence plot (RP). This plot is constructed from the distance matrix *R* with its element $R_{ij}^{\varepsilon }$ given by [21]:

(1) $$R_{ij}^{\varepsilon }=\Theta \;\left(\varepsilon -\|x_{i}-x_{j}\|\right),$$

where *ε* is the threshold value and Θ denotes the heaviside function. Norm ‖ ‖ can be Euclidean, maximal or any other (there we choose Euclidean distance as norm). The RP matrix consists of zeros —white dots (no recurrences) or ones—black dots (occurrence of recurrences). These black spots can be isolated or create diagonal lines (indicating synchronization for some time) or vertical (indicating the duration of the system in a certain state for some time) [26]. An example recurrence plot of selected clinical cases and electrodes is shown in Figure 2.

The qualitative analysis provided by Figure 2 does not clearly distinguish between the patients, although at first glance, it appears that selected EEG signals are characterized by greater regularity in the case of a patient with a disease syndrome. Later, Marwan and others [27,28] and Webber and Zbilut [29] extended the concept of recurrence plots, and on the basis of it, proposed certain measures whose values describe the dynamics of the system in a quantitative way (the recurrence quantification analysis, RQA). First of all, the RQA analysis includes RR, which describes the ability of the system to visit the neighborhood of previous states, i.e., measuring the number of recurrence points according to the formula:

(2) $$\mathrm{RR}=\frac{1}{N^{2}}\overset{N}{\underset{i,j\;\neq \;i}{\sum }}R_{ij}^{\varepsilon }.$$

Quantitative analysis is based on the probability of the occurrence of diagonal *p*(*l*) or vertical *p*(*v*) lines (in this article, we compare the recurrence quantifiers based on statistics of diagonal lines):

(3) $$p\left(l\right)=\frac{P^{\varepsilon }\left(l\right)}{\sum_{x=x_{min}}^{N}P^{\varepsilon }\left(l\right)},$$

where $P^{\varepsilon }\left(l\right)$ denotes the histogram of *l* lengths for a fixed threshold *ε*.

The probability distribution of length of diagonal lines can be interpreted as the average synchronization time, which is more common in a healthy patient. Based on the probability *p*(*l*), subsequent measures like determinism DET, mean of diagonal lines L and maximal diagonal line LMAX can be calculated:

(4) $$\mathrm{DET}=\frac{\sum_{l=l_{min}}^{N}lP^{\varepsilon }\left(l\right)}{\sum_{l=1}^{N}lP^{\varepsilon }\left(l\right)},$$

(5) $$L=\frac{\sum_{l=l_{min}}^{N}lP^{\varepsilon }\left(l\right)}{\sum_{l=1}^{N}P^{\varepsilon }\left(l\right)}$$

(6) $$\mathrm{LMAX}=max\left\{L_{i},\;i=1\ldots n_{l}\right\},$$

where $l_{min}$ denotes the minimal value which should be chosen for a specific dynamical system. To exclude diagonal lines created by a small number of points, we adopted $l_{min}=4$. Determinism DET is a measure of the system’s predictability and determines the ratio of recurrence points forming diagonal lines to all recurrence points. It is worth noting that in the case of complex periodic systems, all points will be included in diagonal lines, because after each period, the system is returning closely to the previous state. L refers to the predictability time of the dynamical system and LMAX to the longest diagonal line, respectively. In our case, we will focus only on indicators based on diagonal lines’ statistics, which are a measure of the periodicity of the system, assuming that they show different values depending on the disease case for different brain regions (electrodes). In the case of synchronization, instead of analyzing point recurrences on the same trajectory, we will analyze recurrence on two trajectories (reconstructed state spaces from individual EEG channels), which will allow the cross recurrence plots (CRP). The previously given recurrence indicators definitions for one trajectory (RQA) can be extended to statistics based on the distance between two trajectories—cross recurrence quantification analysis (CRQA).

In this sense, we understand the recurrence between two points (RR) as synchronization in amplitudes between their corresponding electrodes, the determinism (DET)—as a percentage synchronization factor, L—length of mean diagonal lines—as a mean time of this synchronization, and the maximal diagonal line (LMAX)—as a longest time of synchronization. Values of those quantifiers (corresponding to Figure 2) are presented in Table 1. 

**Figure 3:**
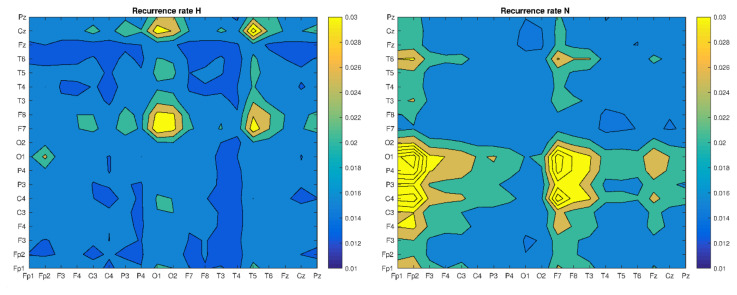

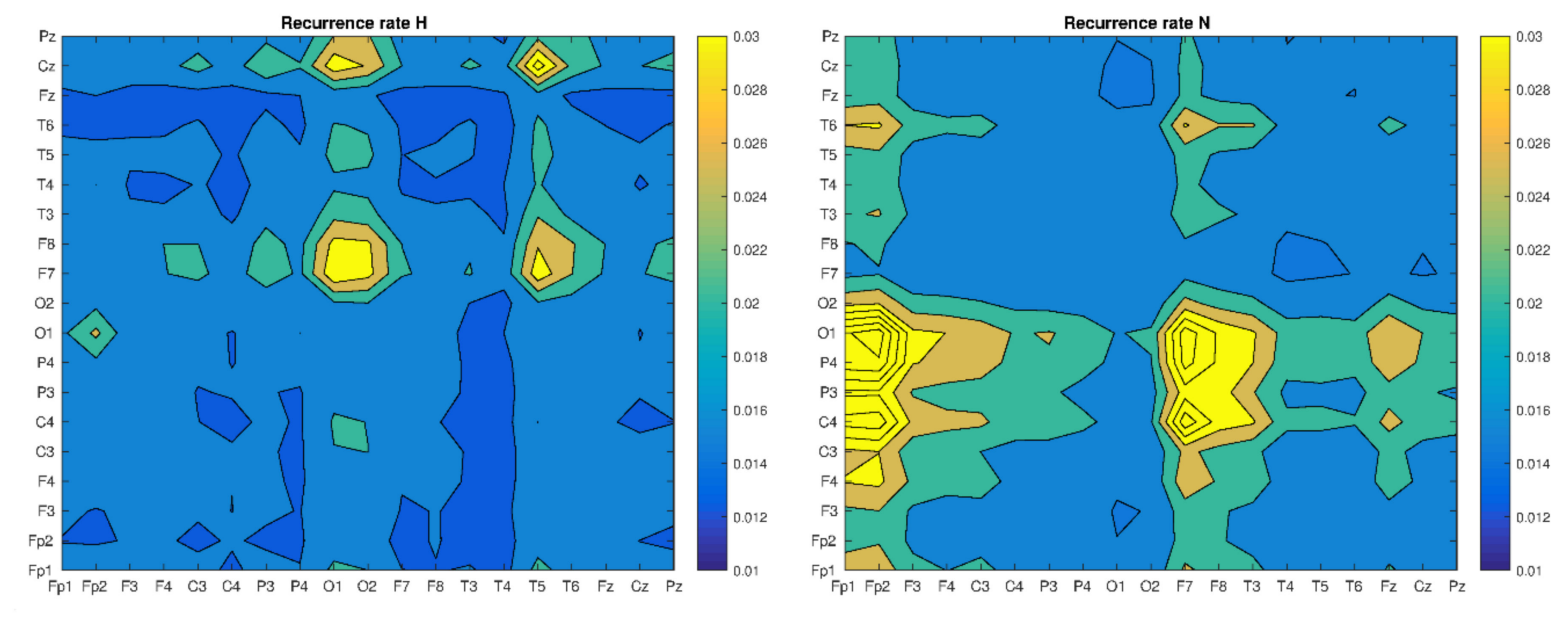
Mean of the Recurrence rate matrix of four trails for all bands: H, N, SN and S, respectively.

When comparing the values of recurrence indicators, a higher value of RR for H, but a comparable mean length of diagonal lines L for H and S, can be seen. Moreover, the longest diagonal line is more than twice as large for the case H. It should be clarified that the choice of the *ε* is critical for the RQA analysis. There are several tips for choosing the optimal threshold value. One of them is the percentage of the standard deviation. The next one is the desired value of recurrence points on the RP. It is recognized that a few percent of recurrence points are sufficient, while not identifying all channels as synchronized. The equal epsilon value *ε* = 1.1 was chosen in this work, in order to compare selected cases with each other. This allowed us to obtain up to 3% recurrence points (Figure 3). Using recurrence analysis with respect to the same dynamic system (in our case, brain dynamics), comparisons between cases can be made either by choosing the *ε* value each time so as to obtain the same number of recurrence points RR (usually without normalizing time series), or using the same value of *ε* (normalizing time series). We adopted the second method, although it should be mentioned that if the number of recurrence points (RR) was the same, the results could be different. However, we believe that the approach taken is appropriate for the cross recurrence analysis, given that the noise level in the measured signals should not change too much.

## 3. Results

**Figure 4:**
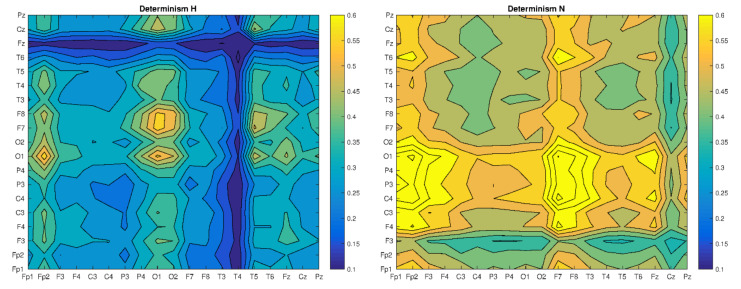

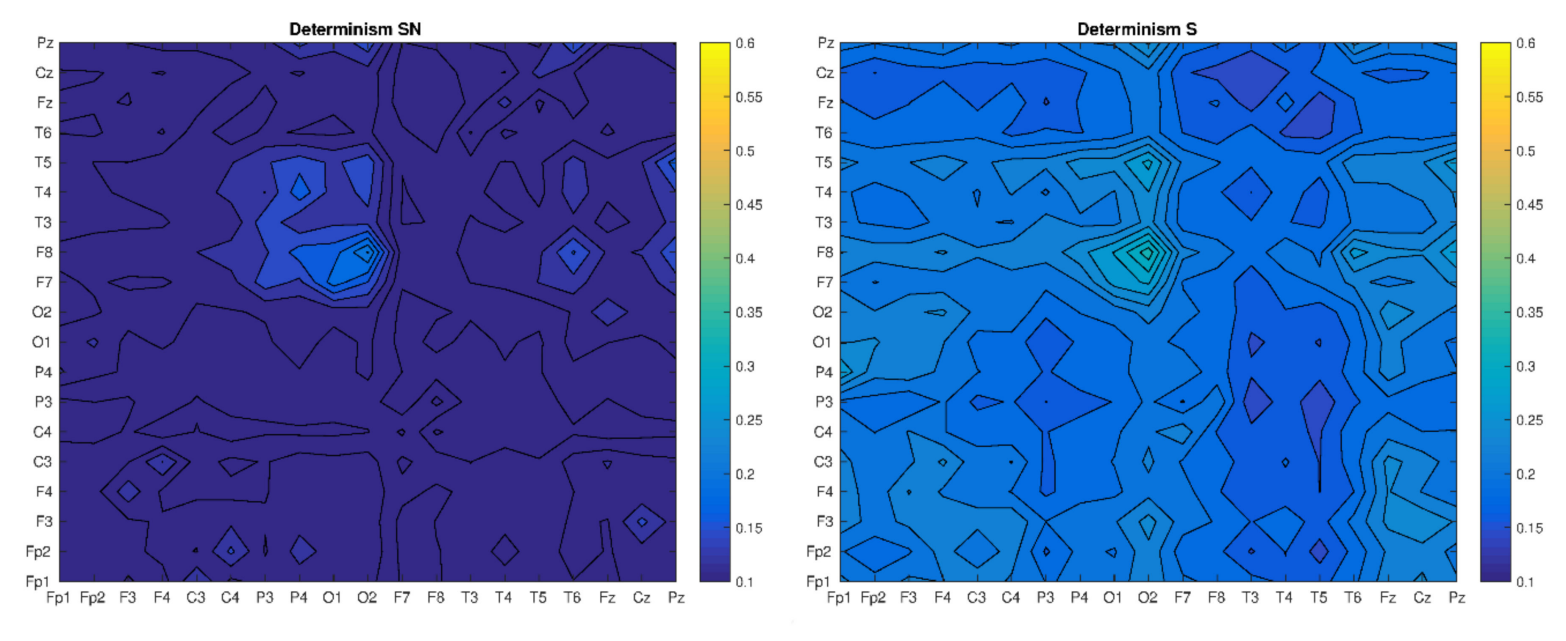
The Determinism matrix of four trails for all bands: H, N, SN and S, respectively.

**Figure 5:**
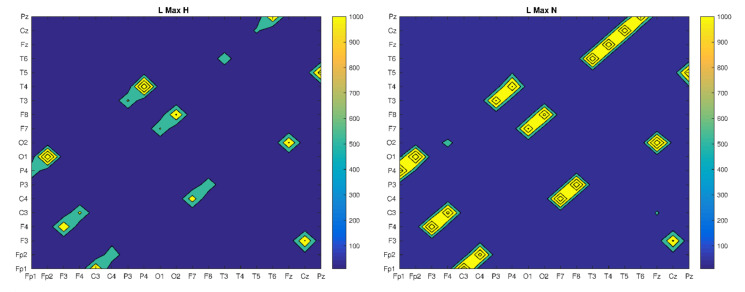

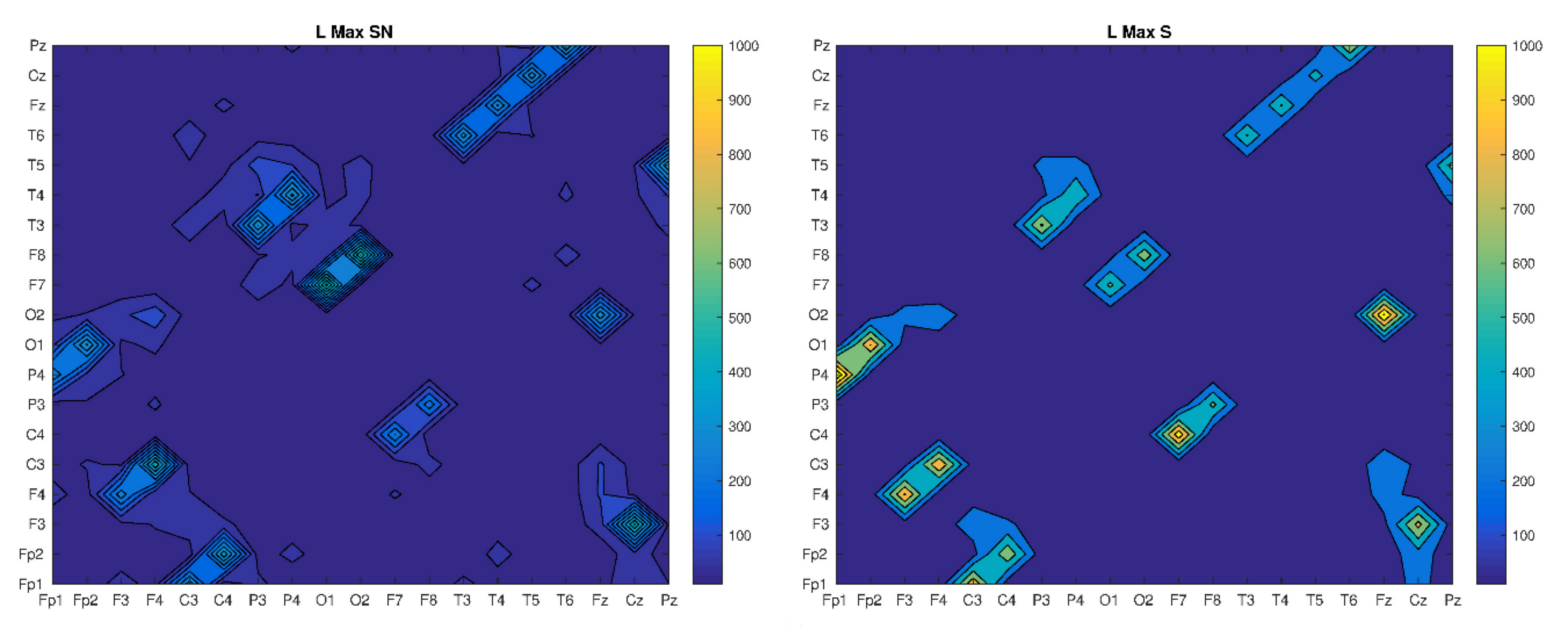
The Maximal length of diagonal lines of four trails for all bands: H, N, SN and S, respectively.

Based on the CRP matrix, we determined and compared the values of recurrence indicators, by looking for some patterns that could help in the classification of patients’ health (Figure 3, Figure 4 and Figure 5).

The analysis in Figure 3 shows the highest synchronization between electrodes H(O1,O2-F7,F8), H(T5,T6-F7,F8), H(O1,O2-Cz) and H(T5,T6-Cz), with a rather homogeneous character for the remaining electrode pairs. Considering the placement of the electrodes on the patients’ head, one can see a certain symmetry between the left and right hemispheres in the occurrence of the highest synchronization. In contrast, more possibilities of synchronization (N(Fp1,Fp2-O1,P3,P4,C4) and N(F7,F8-O1,P3,P4,C4)) can be observed with lose of symmetry. We get a completely different picture in the case of patient SN. In this case, we see rather similar values of the RR for all electrodes with similar pairs as for patient H, but much smaller values. Finally, a large variation in synchronization can also be seen for patient S. The highest RR values were obtained for S(O1,O2-F7,F8,T4,T5), S(Pz-F7,F8) and S(T6-F7,F8), but with varying intensity. It can be said that the RR quantifier varies globally, depending on the type or absence of damage to the patient’s brain. The next stage of our research was to compare the amount of determinism (DET) to the patients considered presented in Figure 4.

Comparing the values of the determinism, one can notice its differentiation in cases (H) and (N) and comparable values for all combinations of SN and N electrode pairs (sample of EEG time series plots between example electrodes placed in Supplementary Material), but the smallest in the last case. It should be noted that in the case of S, the number of synchronizations events (RR) is greater than in the case of H, but the latter shows a greater periodicity (more diagonal lines in the RP). Assuming determinism as the percentage of synchronization, we can observe, globally, completely different scenarios for patients H and N (greater variability) and S and SN (less variability). In the first case, we see areas with a completely different intensity of synchronization. Maximum for healthy patient for H electrodes (O1, O2-O1: F7) and minimal for H (T4-*) and H (*-Fz) electrodes. In turn, for a patient with neurotic disorders, the maximum for N (Fp1: F4-F4: O2) and N (F7: T3-F4: O2) and minimum for electrodes N (*-F3) and N (Cz-*). In this case, you can also see the highest values of the DET indicator. These values for SN and S cases show almost no variation, but are slightly higher for a patient with schizophrenia (S). Thus, in a global scope, the DET values clearly differentiate the patients with schizophrenia from the patients without schizophrenia, regardless of whether there was damage to white matter. Figure 5 shows the longest time of synchronization (LMAX) for all analyzed time series.

In the case of this recurrence indicator, it can be seen that its highest values occur for the same electrode pairs for each patient. However, they differ in the maximum value (the strength of synchronization), which is several times greater for patients without psychiatric diseases (H and N) than for patients diagnosed with psychiatric diseases (SN and S). In the last case, there is also some blur, which is related to the expansion of additional electrode pairs. The LMAX measure can be associated with the measure of the largest Lyapunov exponent, which is a measure of the predictability of a dynamic system. In this case, if the regions of synchronization do not change too much in all the cases analyzed, the strongest synchronization takes place for patients H and N.

## 4. Discussion

In this work, we proposed a non-linear method of recurrence analysis to detect changes in the brain functioning occurring in patients with various disease syndromes, based solely on the EEG time series and their nonlinear properties. It turned out that even such a simple and non-invasive examination of neural activity on the cortical level can distinguish the type of a patient’s disease by means of recurrence quantification analysis. We chose to compare several recurrence indicators based on diagonal lines statistics, whose values we interpreted as synchronization. Our intention was to investigate whether any of the CRQA indicators can be a measure of the complexity of brain work, which is based on the synchronization of registered EEG signals. It turned out that among the selected values, the largest distinction was obtained for determinism (DET), whose values and distribution on the map of electrode connections clearly distinguished a healthy patient (H) from a patient with neurological changes (N), as well as those listed from the other two psychiatric patients (SN, S). In this sense, the complexity of the brain functioning turned out to be the lowest in patients diagnosed with schizophrenia (both with (SN) and without damage to white matter (S)). Since the recurrence analysis is relatively new method to assess functional connectivity based on EEG-recording, we have not dealt with the volume conduction problem, which should be undertaken in future studies. The next direction of research should be the recurrence-based analysis of signals separated into bands, where one might expect a more pronounced differences in the connectivity (synchronization) organization in examined patients. Additionally, as the paper limitation, we should point that we haven’t used filtration algorithms, such as independent component analysis, but the selected signals were thoroughly evaluated by a clinical neurophysiologist with 40 years’ experience, hence, signal samples with artifacts have been removed from the analysis.

Although obtained results seem to be promising, it should be further confirmed with a group analysis containing statistical comparisons. However, our goal was to verify whether the recurrence analysis of resting-state EEG recordings might be applied to diversified patients with different types of neurophysiological disorders, crucial in the development of clinical diagnosis based on objective biological indicators. In this scope, our investigation can be treated as a preliminary to group studies.

## Figures and Tables

**Table 1:** CRQA measures for both selected example electrodes with same *ε* = 1.1.

	RR	DET	L	LMAX
H(F3–F4)	0.035	0.44	6.5	111
S(F3–F4)	0.025	0.25	6.2	47

## Supplementary Materials

The following are available online at https://www.mdpi.com/2076-3425/10/6/380/s1, Figure S1: Sample EEG time series for patient H, Figure S2: EEG time series for patient N, Figure S3: EEG time series for patient SN, Figure S4: EEG time series for patient S.

## References

[1] Andreasen N.C., Nopoulos P., O’Leary D.S., Miller D.D., Wassink T., Flaum M. (1999). Defining the phenotype of schizophrenia: Cognitive dysmetria and its neural mechanisms. Biol. Psychiatry.

[2] Friston K.J. (1998). The disconnection hypothesis. Schizophr. Res..

[3] Krukow P., Jonak K., Karakuła-Juchnowicz H., Podkowiński A., Jonak K., Borys M., Harciarek M. (2018). Disrupted functional connectivity within the left prefrontal cortex and sensorimotor areas predicts impaired cognitive speed in patients with first-episode schizophrenia. Psychiatry Res. Neuroimaging.

[4] Kubicki M., McCarley R., Westin C.-F., Park H.-J., Maier S., Kikinis R. (2007). A review of diffusion tensor imaging studies in schizophrenia. Schizophr. Res..

[5] Hubl D., Koenig T., Strik W., Federspiel A., Kreis R., Boesch C. (2004). Pathways that make voices: White matter changes in auditory hallucinations. Arch. Gen. Psychiatry.

[6] Krukow P., Jonak K., Morylowska-Topolska J., Karakuła-Juchnowicz H. (2007). Specific neuropsychological and neurophysiological dysfunctions of a patients with first-episode schizophrenia and comorbid white matter damage. Acta Neuropsychol..

[7] Baleja-Stawicka I., Kwiecińska E., Kłoszewska I.A.Ł. (2008). Metachromatic leucodystrophy as a cause of dementia and organic delusional syndrome in young adults -a case report. Adv. Psychiatry Neurol..

[8] Lai C.-Y., Scarr S., Udawela M., Everall I., Chen W.J., Dean B. (2016). Biomarkers in schizophrenia: A focus on blood based diagnostics and theranostics. Word J. Psychiatry.

[9] Singer W. (1999). Neuronal synchrony: A versatile code for the definition of relations?. Neuron.

[10] Uhlhass P.J., Singer W. (2010). Abnormal neural oscillations and synchrony in schizophrenia. Nat. Rev. Neurosci..

[11] Jonak K., Krukow P., Karakuła-Juchnowicz H. (2016). Hypercoherence and increased energy of gamma oscillations in patient with first onset of schizophrenia and cerebral white matter damage. Curr. Probl. Psychiatry.

[12] Sakkalis V. (2011). Review of advanced techniques for the estimation of brain connectivity measured with EEG/MEG. Comput. Biol. Med..

[13] Litak G., Syta A., Gajewski J., Jonak J. (2010). Detecting and identifying non-stationary courses in the ripping head power consumption by recurrence plots. Meccanica.

[14] Syta A., Jonak J., Jedliski U., Litak G. (2012). Failure diagnosis of a gear box by recurrences. J. Vib. Acoust. Trans. ASME.

[15] Acharya U.R., Faust O., Kannathal N., Chua T., Laxminarayan S. (2005). Non-linear analysis of EEG signals at various sleep stages. Comput. Methods Programs Biomed..

[16] Rangaprakash D., Pradhan N. (2014). Study of phase synchronization in multichannel seizure EEG using nonlinear recurrence measure. Biomed. Signal Process. Control.

[17] Acharya U.R., Vinitha Sree S., Swapna G., Martis R.J., Suri J.S. (2013). Automated EEG analysis of epilepsy: A review. Knowl. Based Syst..

[18] Ngamga E.J., Bialonski S., Marwan N., Kurths J., Geier C., Lehnertz K. (2016). Evaluation of selected recurrence measures in discriminating pre-ictal and inter-ictal periods from epileptic EEG data. Phys. Lett. Sect. A Gen. At. Solid State Phys..

[19] Stam C.J. (2005). Nonlinear dynamical analysis of EEG and MEG: Review of an emerging field. Clin. Neurophysiol..

[20] Lombardi A., Guccione P., Mascolo L., Taurisano P., Fazio L., Nico G. Combining Graph Analysis and Recurrence Plot on fMRI data. Proceedings of the 2015 IEEE International Symposium on Medical Measurements and Applications (MeMeA) Proceedings.

[21] Millan M.J., Fone K., Steckler T., Horan W.P. (2014). Negative symptoms of schizophrenia: Clinical characteristics, pathophysiological substrates, experimental models and prospects of improved treatment. Eur. Neuropsychopharmacol..

[22] Eckmann J.-P., Kamphorst S.O., Ruelle D. (1987). Recurrence Plots of Dynamical Systems. Europhys. Lett..

[23] Takens F. (1980). Detecting Strange Attractors in Turbulence. Lecture Notes in Mathematics Dynamical Systems and Turbulence, Warwick.

[24] Fraser A.M., Swinney H.L. (1986). Independent coordinates for strange attractors from mutual information. Phys. Rev. A.

[25] Kennel M.B., Abarbanel H.D.I. (2002). False neighbors and false strands: A reliable minimum embedding dimension algorithm. Phys. Rev. E.

[26] Webber C.L., Marwan N. (2005). Recurrence Quantification Analysis–Theory and Best Practices.

[27] Marwan N., Carmenromano M., Thiel M., Kurths J. (2007). Recurrence plots for the analysis of complex systems. Phys. Rep..

[28] Marwan N., Donges J.F., Zou Y., Donner R.V., Kurths J. (2009). Complex network approach for recurrence analysis of time series. Phys. Lett. Sect. A Gen. At. Solid State Phys..

[29] Webber C.L., Zbilut J.P. (1994). Assessing Deterministic Structures in Physiological Systems Using Recurrence Plot Strategies. Bioeng. Approaches Pulm. Physiol. Med..

