# Supplementary material for: The Clinical Application of EEG-Signals Recurrence Analysis as a Measure of Functional Connectivity: Comparative Case Study of Patients with Various Neuropsychiatric Disorders
Source: Brain Sci. 2020 Jun 16;10(6):380. doi: 10.3390/brainsci10060380 (PMC7349203; doi:10.3390/brainsci10060380)
Supplement: Supplementary file 1 [file brainsci-10-00380-s001.zip › brainsci-798550-supplementary.docx]

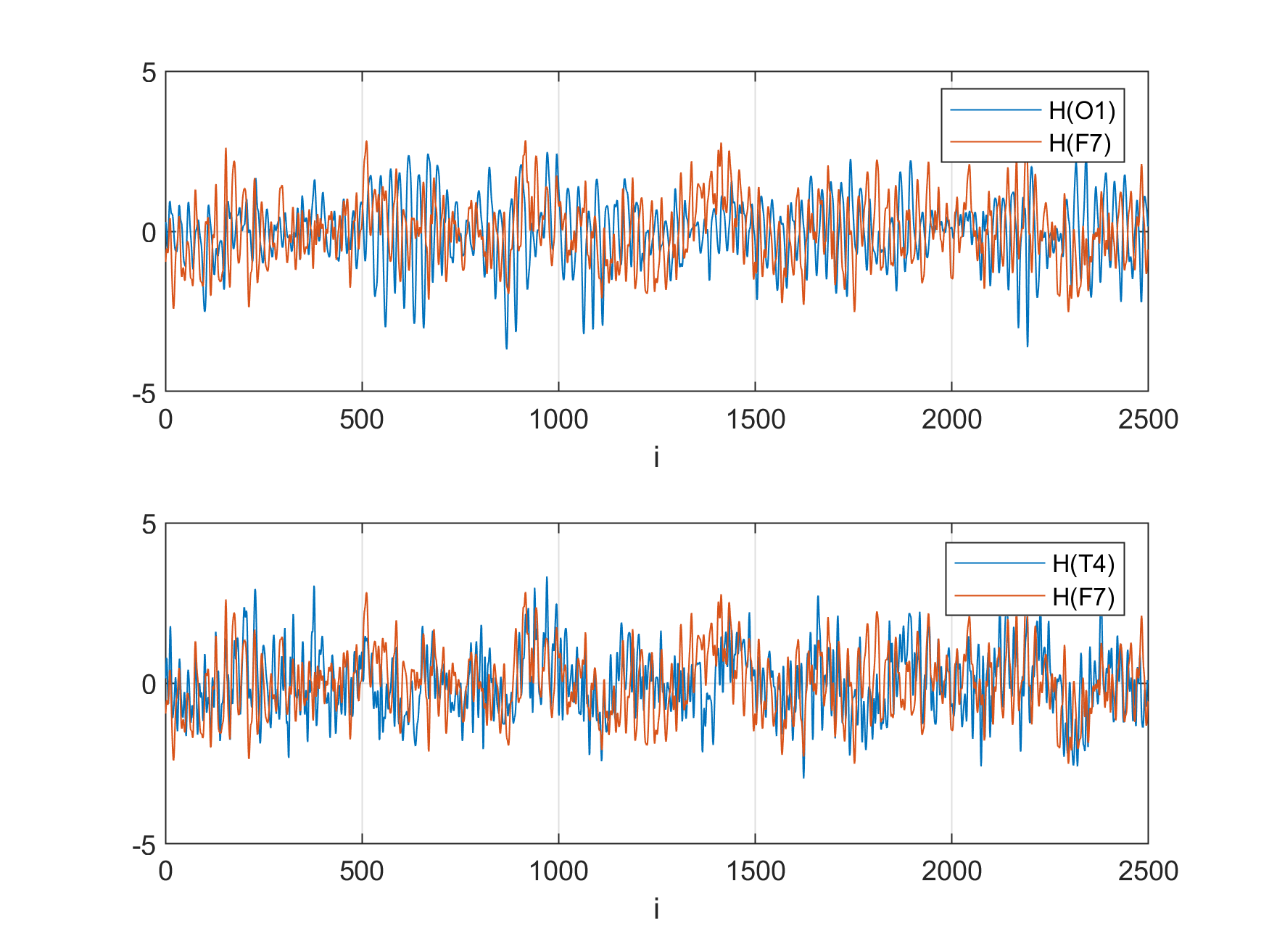


**Figure S1.** Sample EEG time series for patient H (normalized, mean = 0, standard deviation = 1).


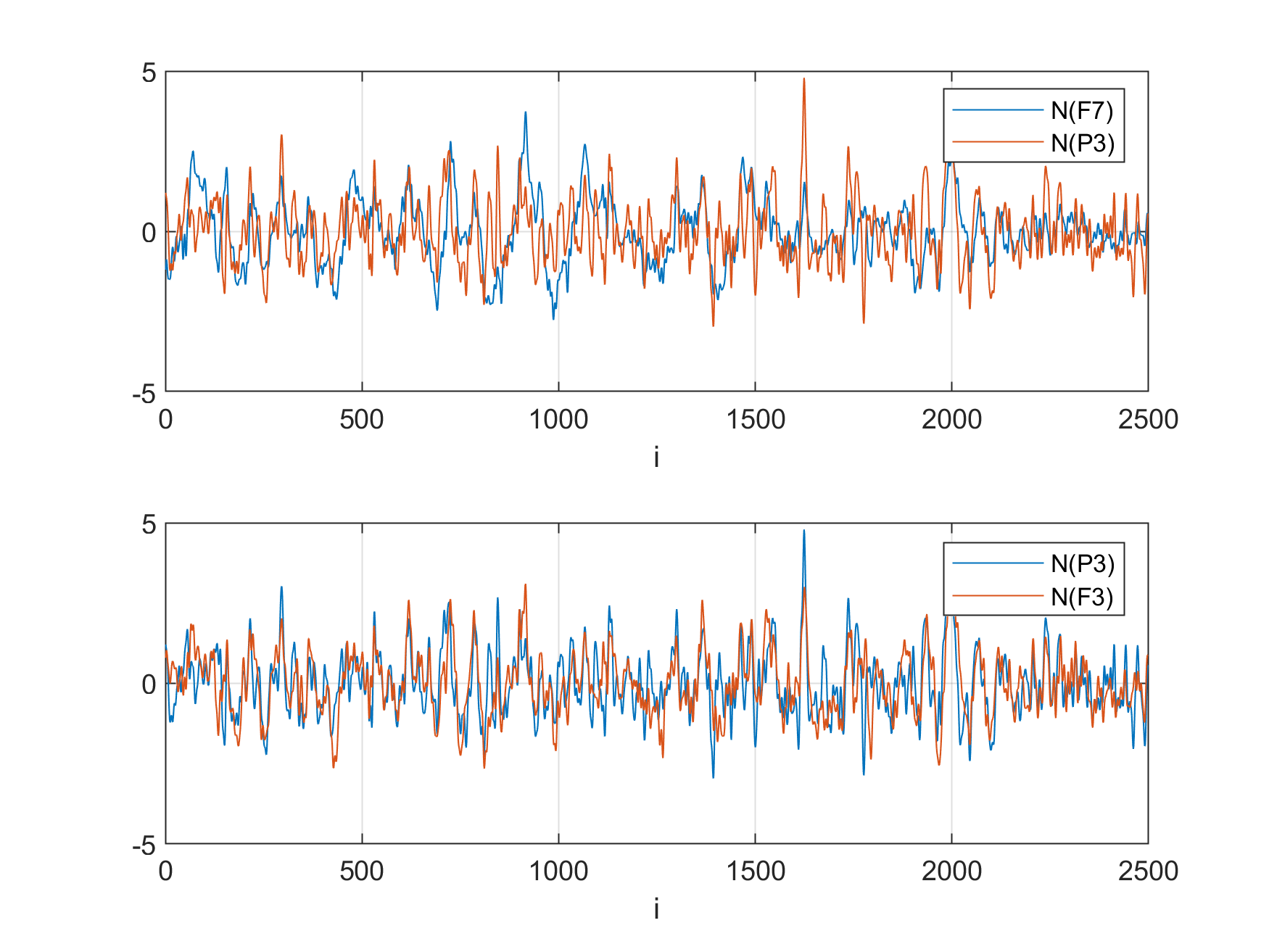


**Figure S2.** EEG time series for patient N (normalized, mean = 0, standard deviation = 1).


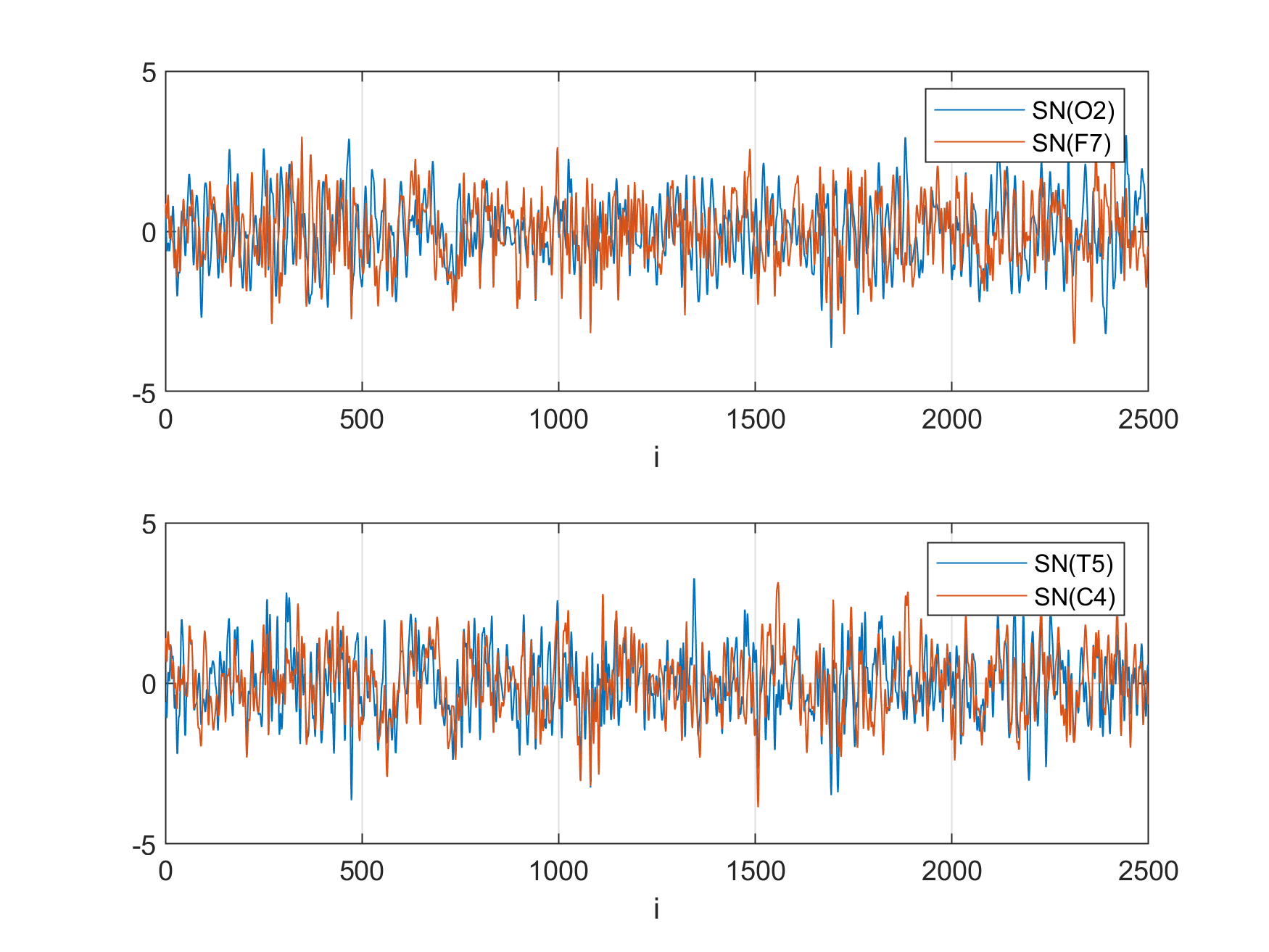


**Figure S3.** EEG time series for patient SN (normalized, mean = 0, standard deviation = 1).


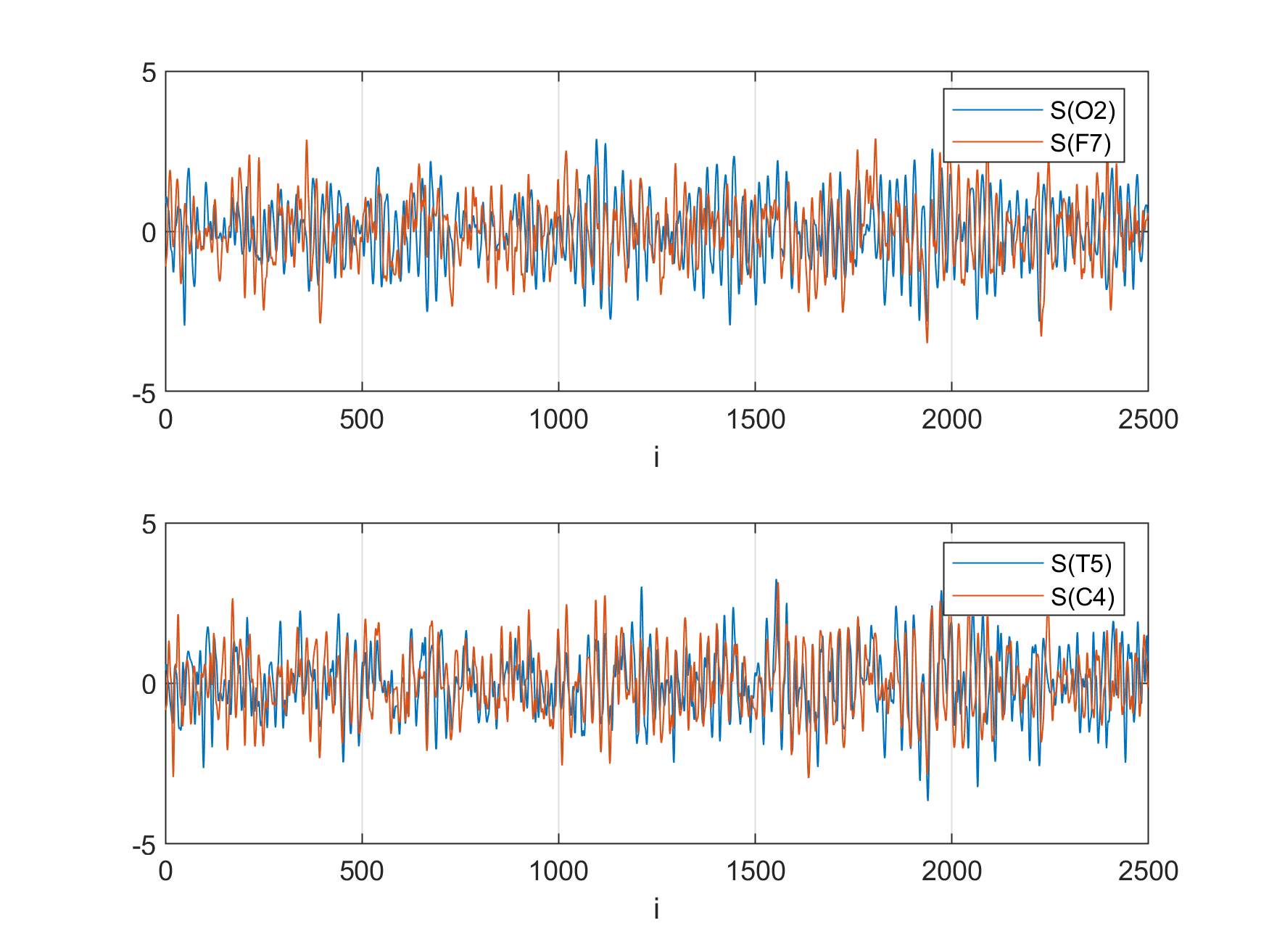


**Figure S4.** EEG time series for patient S (normalized, mean = 0, standard deviation = 1).
